# Time‐Restricted Feeding Preserves Synaptic Function and Modulates Reelin and *α*‐Synuclein in an Acute Amyloid‐*β* Rat Model: A Comparative Study With Alternate‐Day Fasting

**DOI:** 10.1155/jnme/7185647

**Published:** 2026-05-30

**Authors:** Zeynab Maleki, Mohammad Ali Mirshekar, Farzaneh Montazerifar, Bita Etminani, Fateme Keykha

**Affiliations:** ^1^ Student Research Committee, Zahedan University of Medical Sciences, Zahedan, Iran, zaums.ac.ir; ^2^ Clinical Immunology Research Center, Zahedan University of Medical Sciences, Zahedan, Iran, zaums.ac.ir; ^3^ Department of Physiology, School of Medicine, Zahedan University of Medical Sciences, Zahedan, Iran, zaums.ac.ir; ^4^ Genetics of Non-Communicable Diseases Research Center, Zahedan University of Medical Sciences, Zahedan, Iran, zaums.ac.ir; ^5^ Department of Food Sciences and Nutrition, School of Medicine, Zahedan University of Medical Sciences, Zahedan, Iran, zaums.ac.ir

**Keywords:** *α*-synuclein, Alzheimer’s disease, intermittent fasting, rat, reelin

## Abstract

**Introduction:**

Alzheimer’s disease (AD), a major neurodegenerative disorder, is characterized by progressive cognitive decline and the accumulation of amyloid‐beta and tau proteins in the brain. Intermittent fasting (IF) is being explored as a dietary intervention to mitigate AD‐related effects, possibly by modulating factors such as reelin and *α*‐synuclein, which are involved in synaptic function and AD pathology.

**Methods:**

The study included six groups of rats: Ctrl, Ctrl.ADF, Ctrl.TRF, AD, AD.TRF, and AD.ADF. AD was induced by bilateral ICV injections of 5 μL Aβ. To prove fasting, blood glucose levels were assessed with a glucometer. Memory and learning were assessed using the Morris Water Maze (MWM) test, and hippocampal reelin and *α*‐synuclein concentrations were quantified via ELISA. Electrophysiological recordings were analyzed using eProbe software.

**Results:**

TRF was more effective than ADF in improving cognitive function in AD rats, as indicated by a significant increase in TSGQ [*F* (5,40) = 5.590, *p* < 0.01] and swimming speed [*F* (3,21) = 114.3, *p* < 0.01], along with a significant reduction in latency time and path length (*p* < 0.001). Both TRF and ADF significantly increased reelin concentration and neuronal firing rate (*p* < 0.01), whereas only TRF led to a significant decrease in *α*‐synuclein levels.

**Discussion:**

These findings suggest that TRF may enhance spatial memory and reduce *α*‐synuclein accumulation in AD rats, possibly through mechanisms associated with synaptic plasticity and neuroprotection.

## 1. Introduction

Alzheimer’s disease (AD), the most common form of dementia and neurodegenerative disorder, is characterized by a progressive decline in memory and cognitive abilities. It is rapidly becoming one of the most costly, debilitating, and life‐threatening diseases of this century [1, 2].

The World Health Organization (WHO) reported in 2023 that there are over 55 million people with dementia worldwide, with more than 60% of those affected residing in developing countries. Roughly 10 million new dementia cases occur each year [3]. Age‐related dementia and AD are the leading causes of disability in the elderly, and the risk of developing AD increases exponentially with age. By 2050, the prevalence of dementia is expected to rise by 68% in low‐ and middle‐income countries [4].

Progressive memory loss, impaired executive function, and difficulties in performing daily activities are hallmark clinical symptoms of AD. Early manifestations often include subtle behavioral or cognitive changes, impaired recall of newly acquired information, and the onset of language and speech difficulties [4]. The pathological features of AD are classically characterized by the accumulation of amyloid‐beta (Aβ) plaques and phosphorylated tau (p‐Tau), leading to neurofibrillary tangles (NFTs), along with neuronal loss, cerebrovascular amyloidosis, inflammation, and widespread alterations in neural connectivity reported in previous studies [4, 5]. The hippocampus, a brain region critical for memory formation, is among the earliest areas affected by Aβ and tau pathology. Accordingly, deficits in short‐term memory are often observed as early and prominent clinical features of the disease [6].

Reelin, a large extracellular matrix glycoprotein released during embryonic development, plays a crucial role in brain formation and function. Cajal‐Retzius cells express high levels of Reelin, which regulates dendritic growth and neuronal migration. In the adult brain, Reelin is produced by *γ*‐aminobutyric acid (GABA)‐ergic neurons in the cortex and hippocampus, where it significantly influences neural plasticity involved in learning and memory processes [7]. Early‐onset AD can result from pathogenic mutations in three genes: amyloid precursor protein (APP), Presenilin 1 (PSEN1), and Presenilin 2 (PSEN2). Experimental studies suggest that reelin may reduce tau hyperphosphorylation and modulate APP processing, potentially decreasing Aβ production and accumulation [7].

*α*‐synuclein (*α*‐Syn) is a 14‐kDa protein predominantly localized at presynaptic nerve terminals, where it associates with the outer membrane of synaptic vesicles. It plays a critical role in modulating synaptic vesicle trafficking and neurotransmitter release, thereby contributing to the regulation of synaptic transmission in neuronal networks [8]. Pathologically, *α*‐Syn is a major component of Lewy bodies (LBs), the hallmark inclusions of synucleinopathies. Beyond Parkinson’s disease, *α*‐Syn has also been implicated in AD pathology. Experimental evidence suggests that *α*‐Syn may interact with tau and Aβ, influencing their aggregation dynamics and contributing to neurodegenerative cascades. Elevated levels of soluble *α*‐Syn have been detected in AD brains and are correlated with cognitive impairment [9]. The simultaneous presence of Aβ and *α*‐Syn in autopsy is observed in more than 50% of AD patients. Compared to typical AD, these patients exhibit a faster rate of cognitive decline and a shorter lifespan [10].

The causes of aging and strategies to prevent or delay neurodegenerative disorders remain unclear. Dietary interventions, including nutritional supplements and meal timing modifications, may help reduce the risk of neurodegeneration. In addition to cognitive and physical exercise, diet has emerged as an important factor influencing the pathophysiology of neurodegenerative diseases [11]. Increasing attention has been directed toward dietary strategies, as no disease‐modifying treatment for AD or dementia has been established despite extensive research efforts [1, 2]. Caloric restriction (CR) may prevent Alzheimer’s‐related cerebral changes through multiple mechanisms, including neuronal protection and amyloid clearance [12]. There are different types of fasting and CR [13].

Intermittent fasting (IF) has been proposed as a dietary intervention that may attenuate age‐related brain decline and reduce neurodegeneration through mechanisms such as lowering oxidative stress, enhancing mitochondrial efficiency, modulating inflammatory responses, promoting neurogenesis, and supporting synaptic plasticity, as suggested by preclinical studies. IF involves restricting food intake to specific time periods or fasting for entire days. During fasting, water, sugar‐free tea, and coffee are generally permitted [14]. Time‐restricted fasting (TRF; 16:8) is an IF diet that involves a short period of calorie intake time in each 24 h [15]. TRF 16:8 method, receiving food in 8 h and not consuming food in the remaining 16 h [14]. Alternate‐day fasting (ADF) involves fasting for 24 h every other day [14].

From an evolutionary perspective, the nervous system has adapted to periods of food scarcity by utilizing alternative energy substrates. Liver glycogen stores are replenished after each meal, providing about 700–900 calories in the form of glucose, which sustains energetic activity for 10–14 h. In the absence of food intake, these glycogen stores gradually deplete, leading to steadily low blood glucose levels. During prolonged fasting, fatty acids are released from adipose tissue, and the liver converts them into ketone bodies—acetoacetate and *β*‐hydroxybutyrate (BHB)—which enter the bloodstream and are used by neurons as an energy source. Ketone bodies become the brain’s primary energy substrate when glucose availability is limited [16, 17].

During fasting, ketone bodies become a preferred energy substrate for the brain and have been suggested to confer neuroprotective effects against *β*‐amyloid toxicity [18]. The mechanisms by which IF may reduce neuropathology and improve cognitive deficits in AD mice include reducing the enzymatic processing of APP, suppressing inflammation, and enhancing the regulation of neuroprotective stress‐response pathways [19].

The present study aims to investigate the effects of IF regimens on cognitive deficits and the expression of AD‐related proteins, reelin, and *α*‐Syn. While previous research has highlighted the roles of reelin and *α*‐Syn in neurodegeneration, the impact of different IF protocols on these factors has not been thoroughly examined. This study specifically compares TRF and ADF to evaluate their relative efficacy in modulating cognitive function, associated molecular markers, and neuronal firing rates in an acute Aβ rat model.

## 2. Materials and Methods

Reelin and *α*‐Syn assay kits were purchased from ZellBio GmbH (Germany), and Aβ [20–30] was obtained from Kimia Pajouh Dorsa Company (Iran).

Animals: Forty‐eight adults male Wistar rats (2 months old, weighing 200–250 g) were obtained from the Laboratory Animal Research Center of Zahedan University of Medical Sciences (ZAUMS). Two rats were housed per cage, and the room temperature was maintained at 21°C–23°C. Animals had ad libitum access to standard laboratory chow and water. All experimental procedures were conducted in accordance with the guidelines of the Ethics Committee of ZAUMS, Zahedan, Iran (IR.ZAUMS.AEC.1402.020).

### 2.1. Experimental Design

Rats were randomly divided into six equal groups (*n* = 8): (A) Control (Ctrl) received a standard diet. (B) Ctrl + ADF maintained on an ADF regimen for 60 days. (C) Ctrl + TRF maintained on a TRF regimen for 60 days. (D) AD induced via injection of Aβ and fed a standard diet. (E) AD + ADF received ADF for 60 days post‐induction of Aβ. (F) AD + TRF received TRF for 60 days postinduction of Aβ.

To induce an AD–like pathology, amyloid‐beta (Aβ; CAS number: 131602‐53‐4) was used. Aβ in acetate form was dissolved in 10% dimethyl sulfoxide (DMSO) to a final concentration of 5 mg/mL, aliquoted into 10 μL microtubes, and incubated at 37°C for 5–7 days to promote oligomer/fibril formation, as previously described [31, 32].

After anesthetizing the animals with a ketamine/xylazine mixture (90/10 mg/kg body weight), the head was shaved. If the surgical process was prolonged, additional doses were administered to maintain anesthesia. Dexamethasone was administered to prevent brain inflammation, and the animal was positioned in a stereotaxic apparatus. The scalp was then incised, and the bregma and lambda landmarks were identified. Based on the Paxinos and Watson atlas, a small burr hole was drilled above the hippocampal CA1 region (DV = −2.8 mm, ML = −2.2 mm, AP = −3.0 mm). Aβ oligomers were injected bilaterally into the lateral ventricles at a volume of 5 μL per side (total 10 μL) using a Hamilton syringe. The injection was performed slowly, and the needle was left in place for several minutes to ensure proper diffusion. The incision was sutured after the injection.

The rats received standard laboratory chow produced by Behparvar Company (code IVC: 91399051287146, General Department of Animal Husbandry, Tehran Province). The feed composition included corn, wheat, soybean meal, starch, vegetable oil, essential vitamins, and minerals (methionine, lysine, threonine, gluten, choline, salt, sodium bicarbonate, mono‐ or dicalcium phosphate, calcium carbonate, and bentonite). Each bag weighed 25 ± 1 kg, with a metabolizable energy of 2900 kcal/kg. The nutrient composition was as follows:

1. Crude protein: 23%
2. Humidity: max 10%
3. Crude fat: at least 4%
4. Raw fibers: max 4%
5. Lysine: 1.15%
6. Calcium: 1%
7. Threonine: 0.7%
8. Phosphorus: 0.65%
9. Salt: 0.5%
10. Methionine: 0.33%
11. Tryptophan: 0.25%

**FIGURE 1:**
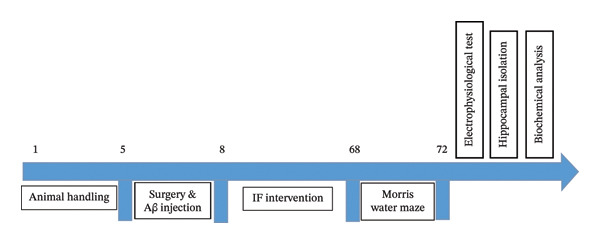
The experimental setup is outlined in a diagram showing the measured variables and their corresponding timeframes.

TRF group: Access to food from 7:00 a.m. to 3:00 p.m. (8 h feeding, 16 h fasting). ADF group: 24 h fasting followed by 24 h feeding (Figure 1). Blood glucose concentrations were measured to confirm fasting. After completion of the 60‐day feeding period, electrophysiological testing was performed.

### 2.2. Morris Water Maze (MWM)

The MWM protocol was conducted following the procedure described by Mirshekar et al. [33]. A circular pool (120 cm in diameter, 50 cm in height) was used to evaluate spatial memory. To assess spatial learning during the training phase, four outcome measures were recorded: (a) mean escape latency, (b) path length to reach the platform, (c) number of entries into the target quadrant during the probe test, and (d) swimming velocity during each trial. Rats underwent the probe retention test one day after the final acquisition session. During this test, the platform was removed, and rats were allowed to swim freely for 60 s [34].

### 2.3. Electrophysiological Study

Rats were anesthetized according to the mentioned method and secured in a stereotaxic frame using Bregma and Lambda as reference points. After drilling, a tungsten microelectrode was implanted into the CA1 region of the hippocampus (Paxinos and Watson coordinates: DV = −2.8, ML = −2.2, AP = −3.0) using a manual microdrive to record single‐unit activity. Neural signals were amplified (gain = 1000), filtered, and isolated based on a signal‐to‐noise ratio greater than 2. Recordings were analyzed as mean firing frequency (spikes/s) over 20 min using *eProbe* software (Tehran, Iran).

### 2.4. Preparing Tissue Samples

Following deep anesthesia, the rats were decapitated. The cerebral tissues were rapidly dissected, and the hippocampus was isolated, blotted dry, weighed, and homogenized in ice‐cold phosphate buffer (10% w/v, pH 7.4). The homogenate was centrifuged at 1000 × *g* for 10 min at 4°C, and the resulting supernatant was collected and stored at −70°C until analysis.

### 2.5. Assessment of Molecular Markers

According to the manufacturer’s instructions, hippocampal reelin and *α*‐Syn contents were measured using enzyme‐linked immunosorbent assay (ELISA) kits (Cat. No: ZB‐1636C‐R9648 for *α*‐Syn and Cat. No: ZB‐16397C‐R9648 for reelin). The hippocampal levels of these biomarkers were quantified using their respective standard curves. The aliquots were then incubated at 37°C for 60 min, and the optical density of the supernatant was measured at 450 nm, expressed as milligrams of tissue protein.

### 2.6. Statistical Evaluation

GraphPad Prism Version 7 software was used to analyze the data. The normality of the data was determined using the Shapiro–Wilk test. One‐way ANOVA was used to analyze blood glucose concentrations, reelin, *α*‐Syn concentrations, and firing rate data. Variables related to the MWM test were analyzed using two‐way repeated measures ANOVA and Tukey’s post hoc test. Values are presented as mean ± SEM. A *p* value < 0.05 was considered statistically significant.

## 3. Results

### 3.1. IF Effects on the Rats’ Path Length During the MWM

Intrahippocampal administration of Aβ resulted in a significant increase in path length during the first three training days of the MWM compared with control animals (*F* [10, 70] = 1.445; ^∗∗^ *p* < 0.001, *p* < 0.0001). Treatment with IF significantly reduced path length relative to the AD group (*F* [10, 70] = 1.445; *p* < 0.01). Moreover, the AD + TRF and AD + ADF groups exhibited shorter path lengths on Days 1–3 of the acquisition trials compared with untreated AD rats (*p* < 0.001, *p* < 0.05), indicating improved memory performance (Figure 2(a)).

FIGURE 2The effect of IF on (a) mean escape latency, (b) path length, (c) total TSGQ in probe trial, and (d) velocity in the Aβ‐induced memory impairments in control, Ctrl.TRF, Ctrl.ADF, AD, AD.TRF, and AD.ADF groups during MWM. (^@^ *p* < 0.05, ^@@^ *p* < 0.01, ^@@@^ *p* < 0.001 vs. control, and ^#^ *p* < 0.05, ^##^ *p* < 0.01, and ^###^ *p* < 0.001 vs. ICV‐Aβ, mean ± SEM (*n* = 8) two‐way ANOVA repeated measurements, followed by Tukey’s post hoc tests).

**Figure:**
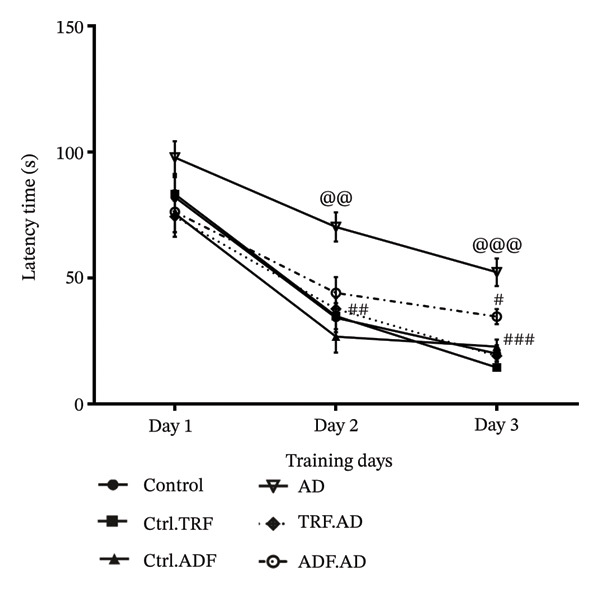
(a)

**Figure:**
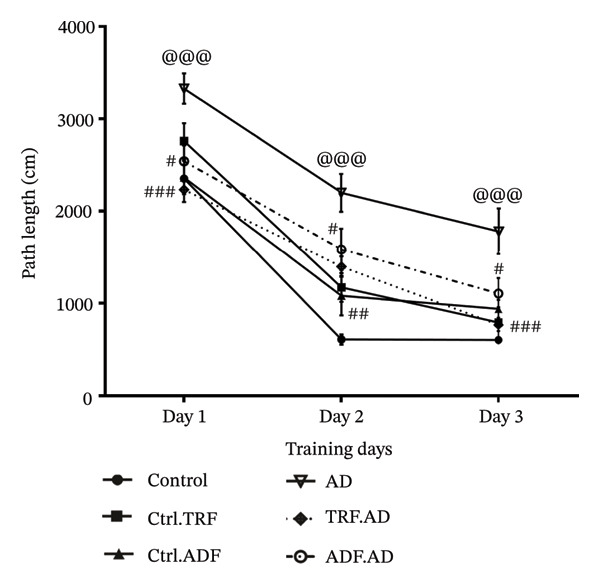
(b)

**Figure:**
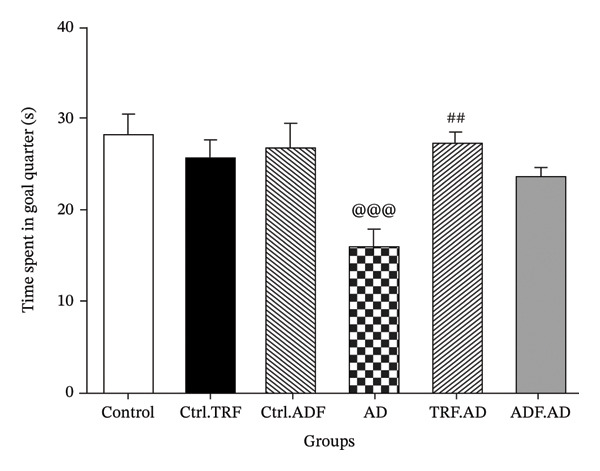
(c)

**Figure:**
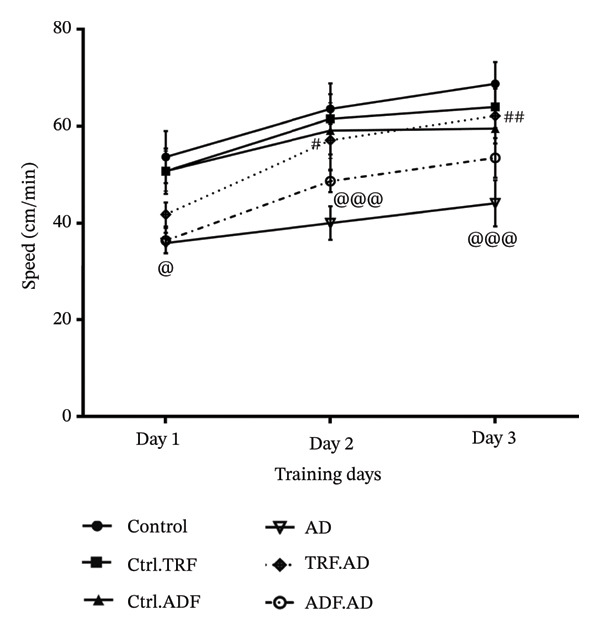
(d)

### 3.2. IF Effects on the Rats’ Escape Latency Means During the MWM

Compared with control rats, AD rats exhibited significantly longer mean escape latencies beginning on Days 2‐3 of the training trials (*F* [10, 84] = 1.261; *p* < 0.01). Notably, AD rats subjected to time‐restricted feeding (AD + TRF) showed a significant reduction in mean escape latency compared with untreated AD rats (*F* [10, 84] = 1.261; *p* < 0.01) (Figure 2(b)).

### 3.3. IF Effects on the Rats’ Time Spent in the Goal Quadrant (TSQG) in the Probe Trial

Spatial memory was assessed using the TSGQ and the duration required to navigate the target area. AD rats exhibited a significant reduction in goal quadrant time compared with controls (*F* [5, 40] = 5.590; *p* < 0.001). Moreover, the duration spent in the goal quadrant was significantly higher in AD + TRF rats than in those administered Aβ (*F* [5, 40] = 5.590; *p* < 0.01) (Figure 2(c)).

### 3.4. IF Therapy Led to a Higher Swimming Velocity in AD Rats

According to Figure 2(d), swimming velocity was significantly reduced in AD rats compared with the control group (*F* [3, 21] = 114.3; *p* < 0.05, *p* < 0.01, *p* < 0.001). However, AD + TRF rats exhibited significantly improved swimming velocity compared with AD rats (*F* [3, 21] = 114.3; *p* < 0.01) (Figure 2).

### 3.5. IF Effects on the Rats’ Blood Glucose

**FIGURE 3:**
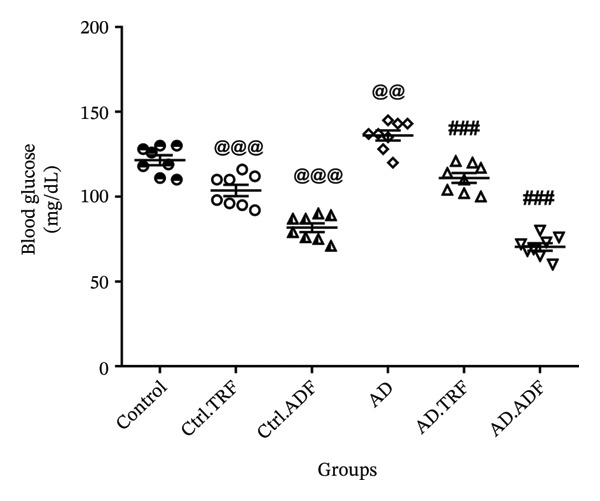
The effect of TRF and ADF regimens on blood glucose concentrations in the Aβ‐induced memory impairments in control, Ctrl.TRF, Ctrl.ADF, AD, AD.TRF, and AD.ADF groups. (^@@^ *p* < 0.01, comparison with control, ^###^ *p* < 0.001, comparison with AD, mean ± SEM (*n* = 8) one‐way ANOVA, followed by Tukey’s post hoc tests).

Blood glucose levels measured by a human glucometer remained stable in the control, Ctrl.TRF, and AD groups under feeding or TRF conditions, while control and AD groups subjected to ADF exhibited a marked reduction in blood glucose on fasting days, which values returning toward baseline on feeding days. The greatest decrease was observed in the ADF.AD group (Figure 3).

### 3.6. Effects of IF on the CA1 Neuronal Firing Rate

Neuronal firing rates in the CA1 region were significantly reduced in AD rats (*p* < 0.001), indicating impaired neuronal communication. Both ADF and TRF interventions increased neuronal firing rates (*p* < 0.01), suggesting a partial recovery of neuronal excitability and synaptic recovery. Although the TRF group showed slightly higher firing rates than the ADF group, the difference was not statistically significant, supporting the beneficial effects of IF on hippocampal function (Figure 4).

FIGURE 4(a) The effect of TRF and ADF regimens on hippocampal firing rate in the Aβ‐induced memory impairments in control, Ctrl.TRF, Ctrl.ADF, AD, AD.TRF, and AD.ADF groups. (b) Raw traces were shown in different groups (^∗∗∗^ *p* < 0.05 vs. control, and ^##^ *p* < 0.01 vs. ICV‐ Aβ, mean ± SEM (*n* = 6). In the B section, tracings of the spontaneous firing pattern of the CA1 location were shown.

**Figure:**
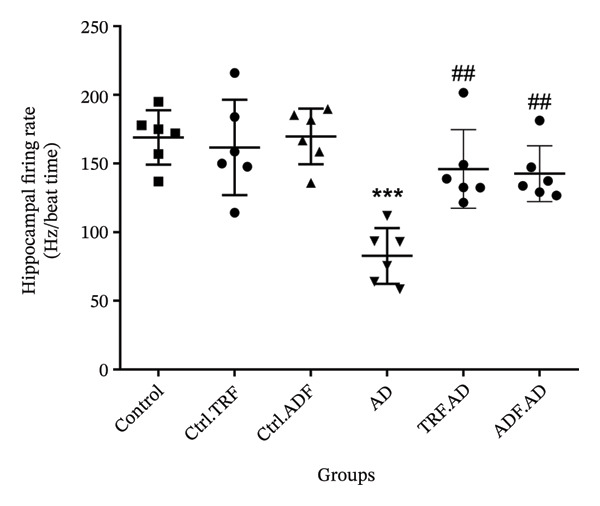
(a)

**Figure:**
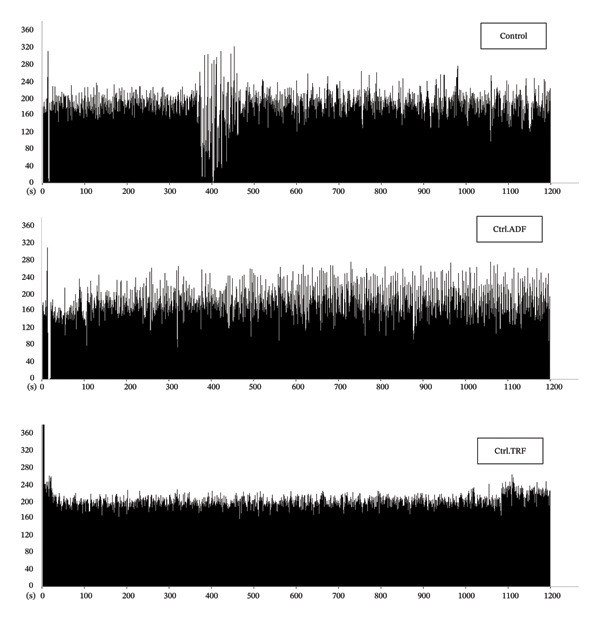
(b)

### 3.7. If’s Effect on *α*‐Syn Levels in Hippocampal Tissue

**FIGURE 5:**
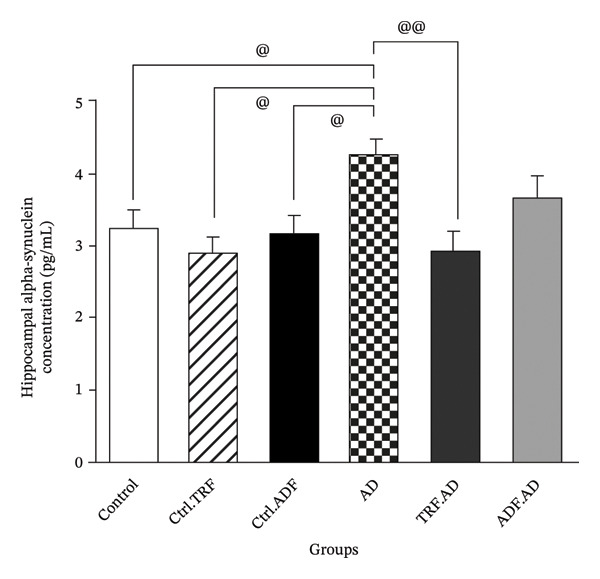
The effect of TRF and ADF regimens on hippocampal *α*‐synuclein concentrations in the Aβ‐induced memory impairments in control, Ctrl.TRF, Ctrl.ADF, AD, AD.TRF, and AD.ADF groups. (^@^ *p* < 0.05, ^@@^ *p* < 0.01, Mean ± SEM (*n* = 8) one‐way ANOVA, followed by Tukey’s post hoc tests).

There was no significant difference in *α*‐Syn concentrations among the Ctrl, Ctrl + TRF, and Ctrl + ADF groups (*p* > 0.05). *α*‐Syn levels were significantly higher in the AD group compared with Ctrl, Ctrl + TRF, and Ctrl + ADF groups (*p* < 0.05). Treatment with ADF and TRF regimens reduced *α*‐Syn levels, with a significant decrease observed only in the AD + TRF group (*p* < 0.01, Figure 5).

### 3.8. If’s Effect on Reelin Levels in Hippocampal Tissue

**FIGURE 6:**
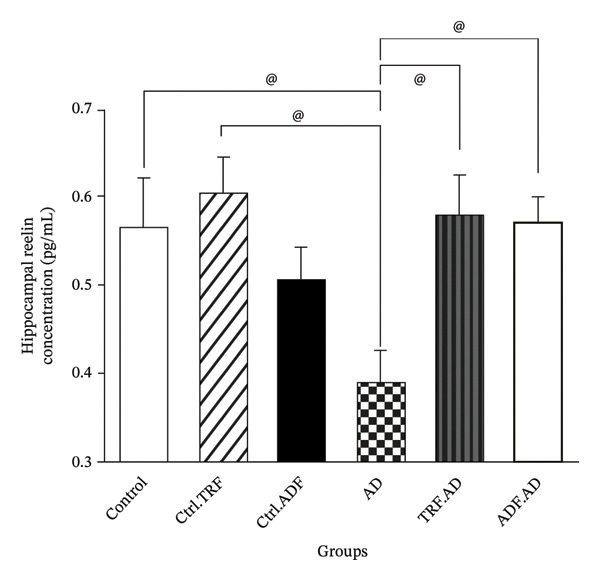
The effect of TRF and ADF regimens on hippocampal reelin concentrations in the Aβ‐induced memory impairments in control, Ctrl.TRF, Ctrl.ADF, AD, AD.TRF, and AD.ADF groups. (^@^ *p* < 0.05, Mean ± SEM (*n* = 8) one‐way ANOVA, followed by Tukey’s post hoc tests).

A significant decrease in reelin concentration was observed in the AD group compared to the Ctrl and Ctrl + TRF groups (*p* < 0.05). There was no statistically significant difference among the Ctrl, Ctrl + TRF, and Ctrl + ADF groups (*p* > 0.05). Treatment with IF regimens increased reelin levels in both AD + TRF and AD + ADF groups, although the changes were not statistically significant (*p* > 0.05, Figure 6).

## 4. Discussion

The present study investigated the effects of IF on cognition and hippocampal levels of reelin and *α*‐Syn in an AD rat model, thereby likely filling a gap in the literature linking IF to these AD‐related proteins. The TRF and ADF diets exerted relatively measurable neuroprotective effects in an acute amyloid‐β model, as evidenced by molecular and behavioral outcomes. The acute Aβ intrahippocampal model was specifically chosen to evaluate the effects of TRF and ADF on Aβ oligomer–induced cognitive and electrophysiological alterations, which are key features of early AD pathology [20, 21, 35]. The study focused on soluble *α*‐Syn and reelin markers, reflecting molecular alterations associated with acute Aβ oligomer exposure and suggesting a potential association between IF and early hippocampal changes. Electrophysiological recordings confirmed that both TRF and ADF increased CA1 neuronal firing rates, indicating enhanced neuronal activity under IF conditions.

The leading theories underlying AD pathogenesis include the amyloid‐β (Aβ) cascade, tau pathology, and cholinergic dysfunction. In addition, AD is associated with a range of pathological processes, such as mitochondrial dysfunction, inflammation, and oxidative stress; however, the precise etiology of the disease remains incompletely understood. Cells within cerebral tissue, including endothelial cells, neurons, astrocytes, and microglia, rely predominantly on glucose as their primary energy substrate to support normal brain function [22].

In this study, stereotaxic injections of toxic Aβ oligomers were used to induce acute neurotoxicity, aiming to model the early synaptic pathology that may precede widespread neuronal loss in AD. The hippocampus was selected as the injection target because it is a primary structure involved in memory processing [23], one of the earliest regions to exhibit metabolic deterioration in the disease [24]. Although this approach allows rapid investigation of Aβ‐induced effects, the exact mechanistic consequences at the synaptic level remain to be fully elucidated.

Due to the marked decline in glucose metabolism, AD is often considered a metabolic neurodegenerative disease. This energy challenge, together with Aβ‐related neurotoxic stress, may contribute to hippocampal vulnerability. Accordingly, exploring IF paradigms that have been associated with metabolic adaptations, including the utilization of alternative energy substrates such as BHB, is of conceptual interest for this energetically compromised region [25]. Early stages of AD are associated with impaired brain glucose utilization, which may contribute to both disease onset and progression [26]. For instance, early AD patients exhibit an approximately 14% reduction in brain glucose uptake compared to control subjects [27]. Reduced glucose uptake may result in an energy deficit that can affect ATP‐dependent cellular processes, potentially contributing to neuronal and regional brain dysfunction [28]. Blood glucose levels increased in the Alzheimer’s group, and both ADF and TRF diets reduced them. After 60 days of IF, blood glucose levels decreased in the ADF‐treated healthy and AD groups compared to the control group. Glucose utilization and mitochondrial function are systemically impaired in people with mild cognitive impairment and AD. Reduced expression of the neuronal glucose transporter Type 3 (GLUT3) in AD has been associated with diminished neuronal glucose uptake and altered regional glucose handling in amyloid‐prone brain areas. Lower GLUT3 levels and elevated tissue glucose have been correlated with increased AD pathology, including amyloid deposition and neurofibrillary changes [25]. In this context, the human brain capacity to utilize ketone bodies—such as BHB, acetoacetate, and acetone—as alternative energy substrates is significantly increased [29]. The transport of ketone bodies across the blood–brain barrier and into neural cells is primarily facilitated by monocarboxylate transporters (MCTs). Notably, this alternative energy pathway appears to remain largely preserved despite impairments in glucose transport [30]. Consistent with this, cerebral ketone utilization has been reported to remain relatively intact in individuals with AD and mild cognitive impairment, even in the presence of reduced glucose metabolism [36]. Ketone bodies have therefore been proposed as potential alternative energy substrates in AD, based on their ability to support cellular energy metabolism under conditions of impaired glucose utilization [37, 38].

Previous studies in AD mouse models have reported that ketone‐based interventions can be associated with improvements in learning, memory, and anxiety‐related behaviors, as well as reductions in amyloid and tau pathology across multiple brain regions [39]. These findings have contributed to growing interest in dietary and metabolic strategies, including IF, as potential approaches for addressing brain energy deficits in AD [22]. In the current work, differences in behavioral outcomes between TRF and ADF were observed; however, attributing these effects to specific metabolic mechanisms, such as differential ketone availability or stability, remains speculative. The applied fasting paradigms were based on established protocols previously reported in the literature [14].

ADF involves cycles of normal feeding days interspersed with fasting days, whereas TRF limits daily food intake to a defined time window, typically 4–8 h [40]. These dietary paradigms differ in their temporal patterns of food availability and have been associated with distinct metabolic states during fasting periods. Prolonged fasting has been reported to reduce hepatic glycogen stores and promote shifts in systemic energy metabolism, including increased reliance on lipid‐derived substrates and ketone body production [22]. ADF led to more visible systemic metabolic changes, including a marked drop in blood glucose. Compared to TRF, no improvement was observed in synaptic proteins or spatial memory. It appears that metabolic responses following beta‐amyloid injection do not necessarily translate into CNS plasticity. On the other hand, TRF is more consistent with the circadian rhythm and may provide more favorable physiological conditions for maintaining synaptic protein balance and cognitive function.

IF paradigms have also been proposed to modulate brain energy metabolism, based on reports indicating enhanced cerebral utilization of alternative energy substrates under fasting conditions [16]. Importantly, neurons affected by AD pathology appear capable of utilizing ketone bodies despite impairments in glucose metabolism, which supports the continued interest in metabolic modulation as a conceptual framework. It was hypothesized that ADF and TRF differentially affect oxidative stress and neurotrophic signaling pathways [41]. Consequently, any interpretation regarding differential metabolic or neuroprotective efficacy between ADF and TRF should be considered exploratory and requires further experimental validation.

Glucose and insulin regulation have been proposed as factors contributing to the metabolic effects associated with TRF. Previous studies in non‐AD populations have reported improvements in insulin sensitivity and glycemic control under TRF paradigms. For example, Che et al. demonstrated that a 10‐h restricted feeding window was associated with improved blood glucose regulation and insulin sensitivity, accompanied by weight loss and reduced reliance on hypoglycemic medications [42]. BHB is a potential mediator of metabolic and neuronal effects under conditions of reduced glucose availability. BHB may be associated with age‐related physiological processes, including aspects of cognitive function [43]. In addition to metabolic substrates, circadian alignment of food intake has been proposed as a factor influencing metabolic health. TRF limits food consumption to specific time windows that coincide with endogenous circadian rhythms, a pattern associated with improvements in glucose metabolism and insulin sensitivity in prior studies [44].

N‐methyl‐D‐aspartate receptors (NMDARs) are known to play important roles in synaptic signaling, and previous studies have reported alterations in the mRNA and protein levels of NMDAR subunits in the brains of AD patients, which have been correlated with cognitive deficits. In particular, the levels of GluN1 and GluN2B subunits have been reported to be reduced in the hippocampus of individuals with AD [45]. IF has been explored in previous studies for its potential effects on neural function and plasticity. Notch signaling has been discussed as a pathway that may contribute to adult hippocampal neurogenesis [46]. IF can influence signaling pathways, including Notch1 and CREB, and modulate markers such as Nestin and BDNF [46]. Reelin, a glycoprotein essential for neuronal positioning and synaptic plasticity, becomes implicated in neurological disorders when dysregulated. It is composed of 3461 amino acids and has a predicted molecular mass of approximately 410 kDa [47]. Previous research has described its interaction with receptors such as very‐low‐density lipoprotein receptor (VLDLR) and apolipoprotein E receptor‐2 (ApoER2), leading to downstream phosphorylation events involving Dab1 and Src family kinases, which may influence NMDAR function [45]. Hippocampal injection of reelin reduced object recognition memory impairments and anxiety‐like behavior in the hippocampus in an animal model of schizophrenia [48]. Similar effects of reelin have been reported in other studies, reversing hippocampal‐dependent synaptic and cognitive dysfunction in a mouse model of Angelman syndrome—a genetic disorder—that disrupts nervous system development, with intraventricular injection of reelin [49]. TRF and ADF regimens with increased reelin concentrations enhanced neuronal firing in hippocampal CA1 neurons. In neuronal plasticity, structural plasticity, and synaptic scaling work cooperatively to maintain neuronal firing rates within a functional range. Thus, while synaptic scaling adjusts the gain of all synapses, structural plasticity changes the total number of synaptic inputs. The interaction of these two mechanisms ensures appropriate neuronal firing rates [50]. Thus, increased neuronal firing rates can indicate improved synaptic structure. Previous studies have indicated that reelin loss can precede the onset of Aβ pathology in the mouse hippocampus and in preclinical human cortical regions [51]. In conditional Ndel1 knockout (CKO) mice, hippocampal injection of reelin protein has been reported to be associated with improvements in spatial learning and memory, potentially linked to alterations in neuronal excitability [52]. Although ELISA quantifies total reelin protein levels, it does not distinguish between full‐length and proteolytically cleaved fragments, which may have differential functional implications. However, these findings were derived from specific animal models, and the relevance of such mechanisms to AD in humans remains to be fully elucidated.

*α*‐Syn is a small, 140‐amino‐acid protein with a molecular weight of approximately 14 kDa. Although it is expressed in peripheral tissues such as the kidneys and blood cells, its highest expression is observed in neuronal presynaptic terminals, including the cerebral cortex, cerebellum, striatum, thalamus, hippocampus, and olfactory bulb [53]. Lewy body (LB) pathology has been reported in a subset of AD cases, and elevated soluble *α*‐Syn levels have been associated with cognitive decline, suggesting potential involvement in neurodegenerative processes [9]. Both *α*‐Syn and *β*‐synuclein are predominantly expressed in the brain and have been linked to synaptic function in previous studies. TRF increased reelin protein levels and modulated *α*‐Syn expression. These molecular changes were associated with a significant enhancement in spatial learning and memory status in the MWM.

It is important to note that protein levels were measured using ELISA, which reflects total protein content and does not distinguish between specific fragments or posttranslational modifications. Therefore, further mechanistic studies are needed to clarify the precise signaling pathways involved.

The role of Aβ in AD is influenced by its interactions with *α*‐Syn. Autopsy studies have reported that over 50% of AD cases exhibit *α*‐Syn pathology alongside Aβ and tau proteins. Immunohistochemical analyses have detected *α*‐Syn within the cores of Aβ plaques, suggesting a potential association with amyloid pathology [54]. In addition, elevated CSF *α*‐Syn levels have been correlated with tau in AD patients [53]. Understanding *α*‐Syn toxicity is vital for developing therapies for synucleinopathies and AD [9]. While these findings provide insight into possible protein–protein interactions in AD, the present study did not investigate the functional consequences of *α*‐Syn in plaque formation or neuronal toxicity.

Limitations of the study include the following: A key limitation is the lack of direct measurement of autophagic flux (e.g., LC3‐II, p62). Although changes in mTOR and neurogenesis pathways suggest altered autophagy, its activation cannot be conclusively confirmed. Future studies should include direct assays such as lysosomal or autophagy flux measurements to validate autophagy’s contribution to the neuroprotective effects of TRF and ADF. The study used an acute Aβ injection model, which reproduces early neurotoxicity but does not represent the chronic progression of human AD. Therefore, the findings are limited to acute Aβ‐induced effects. The mechanistic interpretation is constrained by the lack of measurements of key metabolic intermediates (e.g., BHB, NAD+) and autophagy markers, as well as the absence of histological verification of amyloid plaques and tangles. Long‐term transgenic models and targeted biochemical assays are needed to confirm these mechanisms. Cognitive assessment was limited to the MWM; additional behavioral tests (e.g., Novel Object Recognition, Y‐maze) would provide a broader evaluation of cognitive function. Sex differences were not analyzed, which may affect the generalizability of the findings. Finally, the sample size was relatively small, and future studies with larger cohorts are warranted to improve statistical power and reproducibility.

## Funding

Funding for this research was provided by the research department of Zahedan University of Medical Sciences (grant number: 11201), and all experimental protocols were performed in accordance with the regulations set by the Ethics Committee of Zahedan University of Medical Sciences, Zahedan, Iran (IR.ZAUMS.AEC.1402.020).

## Conflicts of Interest

The authors declare no conflicts of interest.

## Data Availability

The data that support the findings of this study are available on request from the corresponding author. The data are not publicly available due to privacy or ethical restrictions.
